# Sensory stimulation for patients with disorders of consciousness: from stimulation to rehabilitation

**DOI:** 10.3389/fnhum.2014.00616

**Published:** 2014-08-11

**Authors:** Carlo Abbate, Pietro D. Trimarchi, Isabella Basile, Anna Mazzucchi, Guya Devalle

**Affiliations:** ^1^Unità Operativa Complessa di Geriatria, Fondazione I.R.C.C.S. Ca' Granda, Ospedale Maggiore PoliclinicoMilan, Italy; ^2^Nucleo di Accoglienza per Persone in Stato Vegetativo, Fondazione I.R.C.C.S. Don Carlo GnocchiONLUS, Milan, Italy

**Keywords:** sensory stimulation (SS), cognitive rehabilitation, neuropsychological rehabilitation, (un)consciousness, disorders of consciousness (DOC), vegetative state (VS), minimally conscious state (MCS)

## Introduction

Sensory Stimulation (SS) for patients with Disorders of Consciousness (DOC) refers to a corpus of approaches aimed at promoting arousal and behavioral responsiveness by the application of environmental stimuli (Giacino, 1996). Despite the different procedures adopted, the method invariably includes presentation of stimuli which are simple, frequent and repetitive, possibly autobiographical and with emotional content. Moreover, stimuli are administered under multiple sensory channels and with a moderate-to-high intensity. SS is a low invasive, not-dangerous, inexpensive, and simple to apply methodology, and for these reasons, it remains a potentially attractive rehabilitative method (Abbate and Mazzucchi, 2011). However, the theoretical basis of SS has not been clearly formulated in the past, and the method is grounded on general assumptions derived from valid, but out-of-date research findings (i.e., enriched environment as a prevention of sensory deprivation and promotion of synaptic re-innervation and arousal). In addition up until now there is no reliable evidence to support, or rule out, the effectiveness of SS in DOC patients (Lombardi et al., 2002; Lancioni et al., 2010). Thus, even though attractive, SS standard method seems to need a renovation.

Recently a large body of work has improved our knowledge about possible residual cognitive functioning of DOC patients. In particular, neurophysiologic and functional brain imaging studies consistently showed that a subset of DOC patients are able to produce some covert responses (e.g., hand movements), despite the lack of any overt behavioral manifestation (Bekinschtein et al., 2008; Cruse et al., 2012), suggesting a preservation of islands of high-order cognitive functioning (e.g., speech processing, mental imagery, etc.) (Schiff et al., 2002; Owen et al., 2006; Coleman et al., 2007; Owen and Coleman, 2008; Monti et al., 2010). Furthermore, consciousness mechanisms have been recently associated to new notions as distributed information (Tononi, 2004), interacting cortical areas and brain connectivity (Laureys, 2005; Rosanova et al., 2012). Consciousness is viewed as the capacity of a system to integrate information and it seems to depend on the brain's ability to support complex activity patterns distributed among interacting cortical areas (Tononi, 2004; Laureys, 2005; Dehaene and Changeux, 2011; Rosanova et al., 2012; Casali et al., 2013)^1^.

The aim of the present article is to evaluate if the main characteristics of SS method would still be appropriate, taking into account recent research findings and theoretical views elaborated on DOCs. In the case of inadequacies we suggest some possible modifications to the SS method which allow for improvements in light of new findings.

## Main features of SS standard method

### Simplicity

SS programs are based on the idea that to avoid sensory deprivation you should organize an enriched environment which can promote neural plasticity (Di and Schnakers, 2012). However, following the tacit hypothesis that DOC patients have reduced attention capacities and that simple stimulation is less demanding in terms of cognitive processing, several SS protocols used simple and often meaningless stimulations. Yet simple stimulations might not be appropriate considering that DOC patients can be engaged in structured tasks and may have preserved complex responses suggesting islands of preserved high-order cognitive functioning (Lancioni et al., 2008, 2011). Our hypothesis is that these islands could be the target of a SS protocol. If so, a simple and meaningless stimulation could be less efficient in engaging the patient preserved cognition with respect to a more structured and meaningful stimulation. This hypothesis would seem to be supported by a previous fMRI study showing a marked reduced cortical response to linguistically meaningless auditory stimuli with respect to meaningful stimuli in DOC patients (Schiff et al., 2005). Consequently, the use of structured and meaningful stimuli would appear to be a valuable option for SS programs (Table 1).

**Table 1:** **Revision of the main features of the SS standard method and suggestions for possible improvements**.

**SS features**	**Limitations**	**Advantages**	**Possible improvements**
Simplicity	Preserved islands of high-order cognitive processing not engaged		Complex stimulation including structured and meaningful stimuli
Frequency and repetition	Habituation		No repetitive and frequent stimulation
Moderate-to-high intensity	Habituation?	Attention triggered by stimuli with strong energy and sharp onset	Appropriate intensity stimulation, occasionally interspersed with high intensity stimulation
Unimodal stimulation	Attentional sources not captured		Integrated and simultaneous multisensory stimulation
	Preserved islands of high-order cognitive processing not engaged		
Emotional salience		Processing of emotional information prioritized in cognitive system	Emotional stimulation
		Integration promoted	
Autobiographical content		Integration and consciousness promoted	Autobiographical stimulation
		The same advantages as emotional processing	
Input processing	Possible covert answers not advocated		Requests for exhibiting behavioral responses or performing actions
Artificial setting	Absence of emotional salience Absence of autobiographical content		Naturalistic and dynamic actions in real or virtual context
	See limitations for simplicity, repetition, and input processing		

### Frequency/repetition/intensity

SS programs usually consist of presenting a repetitive, frequent, and moderate-to-high intensity simple stimulation. If the aim of SS is to promote patient's behavioral responsiveness and cognitive processing, the concept of Habituation is against these type of protocols (Thompson, 2009). Habituation is in fact defined as a behavioral and neuronal response decrement that results from repeated stimulation (Rankin et al., 2009), therefore a SS procedure organized as repetition of the same stimulus is logically not the best way to maximize the probability of an engagement of cognitive processes during a stimulation protocol. The same conclusion can be held true if we look at the *frequency* characteristic. Classical studies on Habituation suggest that “more frequent stimulation results in more rapid and/or more pronounced response decrement” (Rankin et al., 2009). On the other hand, considering the *intensity* characteristic, some authors suggest that Habituation does not emerge for very intense stimuli (Rankin et al., 2009), while others that it emerges also with intense stimulation (Ritter et al., 1968; Ponce et al., 2011). On this point, it seems relevant to us that the observations that stimuli with strong energy and sharp onsets can activate attention mechanisms can also be seen to lower the threshold for conscious perception (Dehaene et al., 2006).

### Unimodal stimuli

A typical SS approach is defined as multimodal because it usually involves the stimulation of many different sensory modalities (e.g., visual, auditory, tactile, etc.). However, stimuli used are unimodal in nature and sensory channels are stimulated one by one in a standard SS session. Thus, the stimuli in SS are never really multisensory and the method fundamentally provides a serial implementation of different unimodal stimulations. Cognitive neuroscience research in Multisensory integration seems to suggest that unimodal SS is not a valuable option for SS. Several studies suggest that attention tends to orient more easily toward sensory inputs that possess multisensory properties and that this happens automatically (Talsma et al., 2010). Moreover, different lines of research in neurophysiology suggest that brain cortical processing is multisensory not just in associative cortices but also in primary cortices (Ghazanfar and Schroeder, 2006). Taken together, this evidence suggests, from a cognitive and a neurophysiological view, that multisensory stimuli are a valuable option for SS because maybe they are better at capturing attentional sources of DOC patients and in engaging their preserved island of high-order cortical functioning.

### Emotional salience

Stimuli with emotional salience are a valuable option for SS.

Firstly, the processing of emotional information is prioritized in cognitive system (Pessoa, 2005; Vuilleumier, 2005). In particular, emotional stimuli receive privileged access to attention and awareness (capture of attention) (Phelps, 2006). Moreover, sensory processing is enhanced by emotion (Vuilleumier, 2005). In addition, emotion can influence the encoding of to-be-remembered stimuli and arousal is proposed to enhance hippocampal-dependent consolidation (Phelps, 2006). Finally, emotional salience may influence also high-level representations as thoughts and actions (Vuilleumier, 2005). Advantages for emotional against neutral stimuli had been originally found with unpleasant emotional content, however, some similar findings have been recently confirmed also with pleasant content (Lang and Bradley, 2010).

Secondly, emotional processing could favor integration. The notion of the autonomy of emotional processing, for which it would be largely automatic and take place independently of top-down factors such as attention, task context, and conscious awareness, has been recently challenged (Pessoa, 2005, 2008). Studies have in fact shown that amygdalae functions in a manner that is closely tied to top-down factors (Pessoa, 2005, 2008), and that neural circuitry of emotion and cognition interact from early perception to decision making and reasoning (Phelps, 2006). Amygdalae has been hypothesized to be a strong candidate for integrating cognitive and emotional information, considering that is one of the most connected regions of the brain (Pessoa, 2009).

### Autobiographical content

Stimuli with autobiographical content are valuable options for SS. Firstly, the retrieval of autobiographical memories (AM) involves multiple processes, i.e., episodic memory, personal semantic knowledge, visual imagery, emotional processing, self-referential and control executive processes (Cabeza and St Jacques, 2007; Piolino et al., 2009), and accordingly engages a large network of brain regions (predominantly left-lateralized and medial brain regions) (Svoboda et al., 2006). Therefore AM seem to be a proper candidate to promote integration. Secondly, AM are inherently personal and are characterized by varying gradients of emotional content (Svoboda et al., 2006). As a consequence they encompass the advantages from emotional processing. Finally, AM are strictly related to consciousness. In particular, the episodic component of AM are coupled with an high level of consciousness defined “autonoetic” (Tulving, 2002; Markowitsch and Staniloiu, 2011). As a result, stimulating AM could promote consciousness.

### Input processing

Overall SS addresses the input processing. It was in fact limited to stimulate perception, or at most the memory and emotional processing associated with some percepts. On the contrary, the finding of “covert responses” exhibited by some DOC patients supports the view that a new SS should be aimed at stimulating both input and output processing (Bekinschtein et al., 2008; Cruse et al., 2012). If covert responses are possible, we could be authorized to invite the patient to perform actions or complex behaviors during SS even when any overt response is not actually exhibited. Furthermore, in doing so, we could pass from a general *stimulation* that promote arousal to a *rehabilitation* that promotes and reinforces definite behavioral responses by repetitions and exercise. Speaking about brain plasticity, virtually every experience (perception included) has the potential to alter the brain and also producing enduring changes (Kolb and Gibb, 2008). However, it is becoming clear that many experience-dependent changes are highly specific (Kolb and Gibb, 2008). Consequently, a stimulation limited to perception alone could promote only circumscribed brain changes, while larger outcomes would be expected by stimulating both the input and the output processing. Accordingly, *complex-housing* method, which includes social stimulation, SS, and increased motor activity, promotes the greatest benefit to lab animals with injury at any age (Kolb and Gibb, 2008). In addition, a recent theory of action representation regards the *action* as the core of larger representational networks (Hommel and Elsner, 2009). Accordingly, a new SS addressing also the output processing or *actions* could promote integration.

### Artificial setting

The SS typical setting is quite artificial, and looks like a lab where controlled stimuli are administered. This context predisposes to select simple and repetitive stimuli, often without emotional salience and autobiographical content, and aimed to stimulate only the input processing. We believe that naturalistic, dynamic actions in an appropriate context aimed at selecting specific behavioral scripts (e.g., having breakfast with the family) could represent a better choice for a renewed SS (Lancioni et al., 2014). Naturalistic tasks in real or virtual situations involve complex stimuli, call both for input and output processing, and are ideal backgrounds for introducing emotional and autobiographical stimuli. Interestingly, some studies showed that complex and dynamic naturalistic actions can be efficiently assessed by means of a behavioral test (Schwartz et al., 2002) and functional brain imaging (Hugo and Maguire, 2007; Hasson et al., 2009).

## Conclusions

SS standard method is clearly out-of-date and is in need of an overhaul (Abbate and Mazzucchi, 2011). We believe that the new concepts of “covert response” (Bekinschtein et al., 2008), “preserved islands of high-order cognitive functioning” (Owen and Coleman, 2008), and “integration favoring the awareness” (Rosanova et al., 2012) could be a useful theoretical basis for developing a renewed approach to SS. Our revision of SS method, based on the mentioned concepts, has showed that many of its features actually could not facilitate the cognitive processing in DOC patients (Table 1). Conversely, the rationale for applying emotional and autobiographical stimuli has been confirmed. In this article we propose some possible directions for the future based on the concepts of complex stimulation (Di Stefano et al., 2012). This would involve structured and meaningful stimuli that would be delivered to multiple sensory channels in an integrated and simultaneous way. It would address both input and output cognitive processing and involve dynamic and naturalistic actions that would avoid meaningless repetitive and frequent stimulations (Lancioni et al., 2014). This would, therefore, include stimulations with proper intensity which would be occasionally interspersed with intense stimuli. All of these actions would maintain the valid aspects of emotional salience and autobiographical relevance.

### Conflict of interest statement

The authors declare that the research was conducted in the absence of any commercial or financial relationships that could be construed as a potential conflict of interest.

## Footnotes

^1^In the text we'll use the broad term “integration” to address indistinctly these last concepts.
